# Innate stimulation of B cells *ex vivo* enhances antibody secretion and identifies tumour-reactive antibodies from cancer patients

**DOI:** 10.1093/cei/uxab005

**Published:** 2021-12-05

**Authors:** Panagiotis Karagiannis, Isabel Correa, Jitesh Chauhan, Anthony Cheung, Diana Dominguez-Rodriguez, Manuela Terranova-Barberio, Robert J Harris, Silvia Crescioli, James Spicer, Carsten Bokemeyer, Katie E Lacy, Sophia N Karagiannis

**Affiliations:** 1 Department of Oncology, Haematology and Bone Marrow Transplantation with Section of Pneumology, University Medical Centre Hamburg-Eppendorf, Hamburg, Germany; 2 St. John’s Institute of Dermatology, School of Basic and Medical Biosciences, King’s College London, Guy’s Hospital, London, UK; 3 Breast Cancer Now Research Unit, School of Cancer and Pharmaceutical Sciences, King’s College London, Guy’s Cancer Centre, London, UK; 4 School of Cancer and Pharmaceutical Sciences, King’s College London, Guy’s Hospital, London, UK

**Keywords:** tumour immunology, antibodies, B cells, cell differentiation, cell proliferation

## Abstract

Human B cells and their expressed antibodies are crucial in conferring immune protection. Identifying pathogen-specific antibodies following infection is possible due to enhanced humoral immunity against well-described molecules on the pathogen surface. However, screening for cancer-reactive antibodies remains challenging since target antigens are often not identified *a priori* and the frequency of circulating B cells recognizing cancer cells is likely very low. We investigated whether combined e*x vivo* culture of human B cells with three innate stimuli, interleukin-17 (IL-17), B-cell activation factor (BAFF), and the toll-like receptor 9 (TLR-9) agonist DNA motif CpG ODN 2006 (CpG), each known to activate B cells through different signalling pathways, promote cell activation, proliferation, and antibody production. Combined IL-17+BAFF+CpG prolonged B-cell survival and increased proliferation compared with single stimuli. IL-17+BAFF+CpG triggered higher IgG secretion, likely by activating differentiated, memory and class-switched CD19^+^CD20^+^CD27^+^IgD^-^ B cells. Regardless of anti-FOLR antibody seropositive status, IL-17+BAFF+CpG combined with a monovalent tumour-associated antigen (folate receptor alpha [FOLR]) led to secreted antibodies recognizing the antigen and the antigen-expressing IGROV1 cancer cells. In a seropositive individual, FOLR stimulation favoured class-switched memory B-cell precursors (CD27^-^CD38^-^IgD^-^), class-switched memory B cells and anti-FOLR antibody production, while IL-17+BAFF+CpG combined with FOLR, promoted class-switched memory B-cell precursors and antibody-secreting (CD138+IgD-) plasma cells. Furthermore, IL-17+BAFF+CpG stimulation of peripheral blood B cells from patients with melanoma revealed tumour cell-reactive antibodies in culture supernatants. These findings suggest that innate signals stimulate B-cell survival and antibody production and may help identify low-frequency antigen-reactive humoral responses.

## Background

B cells respond to antigen recognition but are also involved in a network of interactions with their environment, influencing B-cell survival, proliferation, maturation, class-switching, and antibody expression. Despite a heightened response at the time of antigen challenge, specific long-term antibody-expressing B-cell clones—even after vaccination—might have a frequency of 1–3% or below in peripheral blood [1]. In the context of cancer, understanding how B cells and the antibodies (Ab) they produce are influenced by the presence of tumour cells, as well as exploiting this knowledge to develop novel strategies to combat solid tumours, are still not sufficiently understood.

B cells extracted from peripheral blood or tissues [2–4] have been stimulated *ex vivo* by certain cytokines such as IL-21, IL-17, IL-2, the B-cell activation factor (BAFF) [5–8], pattern recognition antigens that mimic T-cell-independent signals like R848, CpG ODN 2006 [9, 10], and via CD40 ligation [11]. Activation through the engagement of toll-like receptors (TLRs), expressed by a large spectrum of B-cell subtypes, by several agonists can elicit B-cell activation independently of antigen recognition. Particularly, TLR-9 may be expressed by 78% of all CD19^+^ and 20% of CD27^+^ memory B cells. In response to TLR-9, both naive and memory B cells can secrete IL-6, which may act in an autocrine manner by driving B-cell differentiation into antibody-secreting cells [12]. Furthermore, the growth factor BAFF, a member of the TNF cytokine family, may support B-cell maturation. BAFF has three known receptors: BAFF-R, BCMA, and TACI, all expressed on the cell surface at different stages of B-cell development. The interaction between BAFF and its receptors may support B-cell development; for example, BAFF-R plays a particular role in follicular B-cell survival, as well as retention of antibody responses during long-term memory. Interestingly, studies in animal models suggest that BAFF may also contribute to the induction of autoimmunity. These properties may render BAFF a powerful stimulus for B-cell activation and survival [13]. In addition, the cytokine IL-17 normally associated with autoimmune disorders, may have a synergistic effect with BAFF in promoting B-cell survival, proliferation and immunoglobulin secretion, through activation of transcription factors and anti-apoptotic proteins [6, 14]. IL-17 has been shown to enhance class switching *in vitro* more prominently than IFN-γ and can directly induce antigen-experienced memory B-cell differentiation without secondary T-cell signals. These innate signals may enable *ex vivo* cultured B cells to produce mature antibodies.

In this study, we explored whether combined stimulation with IL-17, BAFF, and CpG may support human B-cell activation and antibody production *ex vivo* and whether stimulation can aid the identification of low-frequency antibodies.

## Methods

### Cell culture

The melanoma cell line A375, (CRL-1619, ATCC) and the ovarian cancer cell line IGROV1 (a kind gift of Dr S. Canevari) were grown in Dulbecco’s Modified Eagles Media (DMEM) (Gibco, Eugene, USA), 10% FCS, 2 mM L-glutamine, penicillin (5000 U/ml), and streptomycin (100 µg/ml). Primary human melanocytes (ATCC, PCS-2000–012) were grown in Dermal Cell Basal Medium (ATCC) and supplemented with the Melanocyte Growth Kit (ATCC). B95-8 marmoset blood leukocyte cell line which secrete Epstein-Barr virus (EBV) was grown in RPMI 1640 medium, 10% FCS, 2 mM L-glutamine, penicillin (5000 U/ml), and streptomycin (100 µg/ml). All cells were maintained in a 5% CO_2_ humidified incubator at 37°C.

### B-cell isolation and activation

B cells were isolated from melanoma patient and healthy volunteer peripheral blood by negative selection using RosetteSep^®^ B cell enrichment cocktail (Stem Cell Technologies) according to the manufacturer’s instructions. Simultaneously, peripheral blood mononuclear cells (PBMC) were isolated using Ficoll (GE Healthcare) gradient centrifugation, irradiated at 30 Gy and used as feeder cells. After isolation, B cells were plated at 200 cells/well, unless otherwise stated, on U-bottom micro-plates (Nunc) along with 2 × 10^4^ autologous irradiated PBMC. B-cell cultures were grown in RPMI 1640 medium supplemented with 10% FCS, 1% penicillin-streptomycin, 30% supernatant collected from Epstein Barr Virus (EBV) producing B95-8 cells, and the following supplements were added alone or in combination: 2.5 ng/ml CpG 2006 ODN (Operon), 100 ng/ml human recombinant B-cell activation factor (BAFF, Peprotech), 1 ng/ml IL-17 (R&D Systems), 2.5 ng/ml GpC (Operon), monovalent recombinant folate receptor alpha (FOLR) (BD Europe Systems Ltd.). For identification of single antigen-reactive B cells derived from melanoma patient blood, cells were either selected immediately for single cell sorting by recognition of antigens conjugated on fluorescent beads, or after *ex vivo* culture with IL-17+BAFF+CpG for 7 days (densities: 10,000–40,000 cells/well), prior to single cell sorting by recognition of antigens conjugated on fluorescent beads, as previously described [3].

In order to screen antibodies against folate receptor alpha B cells were plated on 96 U-bottom plates at 10,000 cells/well together with 50,000 irradiated autologous PBMCs in 200 µl of 10% FCS, RPMI with Glutamax and Pen/Strep. Cells were incubated in medium alone, medium supplemented with 1 µg/ml recombinant folate receptor alfa (FOLR) (R and D Systems), medium supplemented with a mix of CpG (2.5 µg/ml, InvivoGen), human recombinant BAFF (100 ng/ml, Preprotech), and human recombinant IL-17 (1 ng/ml, Preprotech) or medium supplemented with CpG, BAFF, and IL-17 plus 1 µg/ml of FOLR. Control wells with feeders only were plated for the four conditions.

### CFSE proliferation assay

Isolated B cells were labelled with 1 μM carboxyfluorescein succinimidyl ester (CFSE; Thermo Fisher) at 37°C for 10 minutes, then washed twice with RPMI 1640 containing 10% foetal calf serum. Subsequently, cells were co-cultured using different feeder cells or innate stimuli in combination with feeder cells. On day 3, CFSE profiles of CD19+ DAPI- cells were evaluated by flow cytometry. Cell divisions and proliferation index were determined using the Proliferation Wizard algorithm FlowJo v10 (BD Bioscience). The proliferation index is defined as the sum of the cells in all generations divided by the computed number of original parent cells present at the start of the experiments.

### Flow cytometry staining and analysis

Freshly isolated or *ex vivo* cultured human cells were washed in PBS with 5% bovine serum (FACS buffer) and subsequently stained for 30 minutes at 4°C with fluorophore conjugated antibodies against CD19, CD20, CD22, CD27, or IgD (all BD Biosciences). Cells were then washed twice in FACS buffer and analysed on a FACS Canto2 (BD Biosciences). Where appropriate dead cells were excluded using 4,6-Diamidin-2-phenylindol (DAPI; Thermo Fisher).

To analyse B cells after 9–11 days in culture, plates were spun at 400×*g* for 3 minutes, supernatants were taken for analysis by ELISA and cells in pellets were re-suspended and pooled for each condition. Cells were washed with PBS and stained with NIR fixable live/dead dye (Molecular probes) for 15 minutes at room temperature and washed with FACS buffer (2%BSA, 2mMEDTA, PBS). Live/dead staining was followed by an incubation with Fc Block reagent (BioLegend) for 10 minutes at RT and finally staining with a mix of labelled antibodies: CD45-BV785, CD19-FITC, CD27-BV711, CD38-APC, CD138-PE, and IgD-BV421 (all BioLegend) following the manufacturers recommendations for 30 minutes at 4C. Stained cells were washed and fixed with 2% PFA in PBS for 10 minutes at RT. Stained cells were analysed in a Beckton Dickinson Fortessa with 355, 405, 488, 561, and 633nm lasers.

### Flow cytometry data analysis

Live single CD45+ CD19+ cells were manually gated and clustered into groups of phenotypically similar cells based on the expression of a combination of 4 markers (CD38, CD27, CD138, and IgD), using clustering algorithm PhenoGraph as implemented in R (available at https://github.com/JinmiaoChenLab/Rphenograph). The k-neighbors value was selected according to the square root of the number of total events and adjusted to optimize the clustering (*K* = 150). Of the resulting 20 clusters, some clusters were aggregated into a larger group following the hierarchical clustering (Euclidean distance) of their median integrated marker intensities, as shown in the heatmap. For visualization, high-dimensional single cell data were reduced to two dimensions using the t-SNE algorithm (as implemented in the FlowJo software, BD Bioscience, version 10.7.1), where each cluster is represented by a different colour on the map. An heatmap was generated using a R script to identify each cluster. The images (heatmap and tSNE map) report a final number of 12 clusters.

### ELISA

Total IgG secreted in B-cell culture supernatants was measured using a sandwich Enzyme-linked Immunosorbent Assay (ELISA). Briefly 96-well Maxisorp flat bottom plates (Nunc) were incubated overnight at 4°C with a goat anti-human IgG (AbDSerotec) diluted 1:1000 in coating buffer (Carbonate-Bicarbonate Buffer, pH 9.5). The following day, the coating buffer was removed, and plates were blocked with 2% non-fat milk solution in PBS, 0.05% Tween 20 (PBST) for 1 hour on an orbital shaker. Plates were then treated with either supernatants removed from B lymphocyte cultures or different concentrations of a human IgG control antibody (Sigma), from which the standard curve was derived. Plates were incubated for 2 hours at room temperature on an orbital shaker, and bound antibodies were detected with horseradish peroxidase-labelled goat anti-human IgG Fc-specific antibody diluted 1:1000 in PBST (Sigma) for 45 minutes at room temperature on an orbital shaker. After washing of unbound antibody, colour reaction was developed with o-Phenylenediamine dihydrochloride (OPD, Sigma) and OD was measured in an ELISA reader (BMG Labtech) at 492 nm (reference wavelength, 650 nm). Anti-folate receptor alpha IgG ELISA was performed as previously described. The lower detection levels of quantification were 3.125 and 0.625 ng/ml, respectively for 1:5 dilution and neat samples [15, 16].

### Cell-based ELISA

Briefly, A375, IGROV-1 cancer cells or primary human melanocytes were seeded at 3 × 10^4^ cells per well on 96-well flat bottom plates (Corning, Salt Lake City, USA) and grown at 37°C in a humidified incubator until cells reached confluence. Cells were then fixed in 0.5% formaldehyde, HBSS, and plates were stored at −80°C until use. To screen for tumour cell-reactive antibodies secreted by human B cells, plates were defrosted and blocked with a 5% non-fat milk solution for 2 hours at room temperature. Supernatants from B lymphocyte cultures were then added to single wells and plates were incubated for 90 minutes at room temperature on an orbital shaker. Plates were then washed three times with 200 µl per well PBST. Binding of antibodies to surface antigens on tumour cells and melanocytes was detected following a 45-minute incubation with 0.8 μg/ml horseradish peroxidase-conjugated F(ab)’2 Fc-specific antibody (goat) (Jackson ImmunoResearch) at room temperature on an orbital shaker. After a wash to remove any unbound antibody, a colour reaction was developed with o-Phenylenediamine dihydrochloride (Sigma), and OD was measured on an ELISA reader (BMG Labtech) at 492 nm (reference wavelength, 650 nm). Trastuzumab (IgG_1_, Roche) or a non-specific human IgG_1_ (Jackson ImmunoResearch) were used as positive and negative control antibodies, respectively. Positive cultures were defined as having an OD value above 75% of the OD of the positive control antibody.

### Statistical analysis

Statistical analyses were performed using GraphPad Prism Version 6. After analysing for normality using the D’Agostino Normality Test, not normally distributed samples were compared using the Mann–Whitney *U* test. A *P* value < 0.05 was considered statistically significant.

## Results

**Fig. 1:**
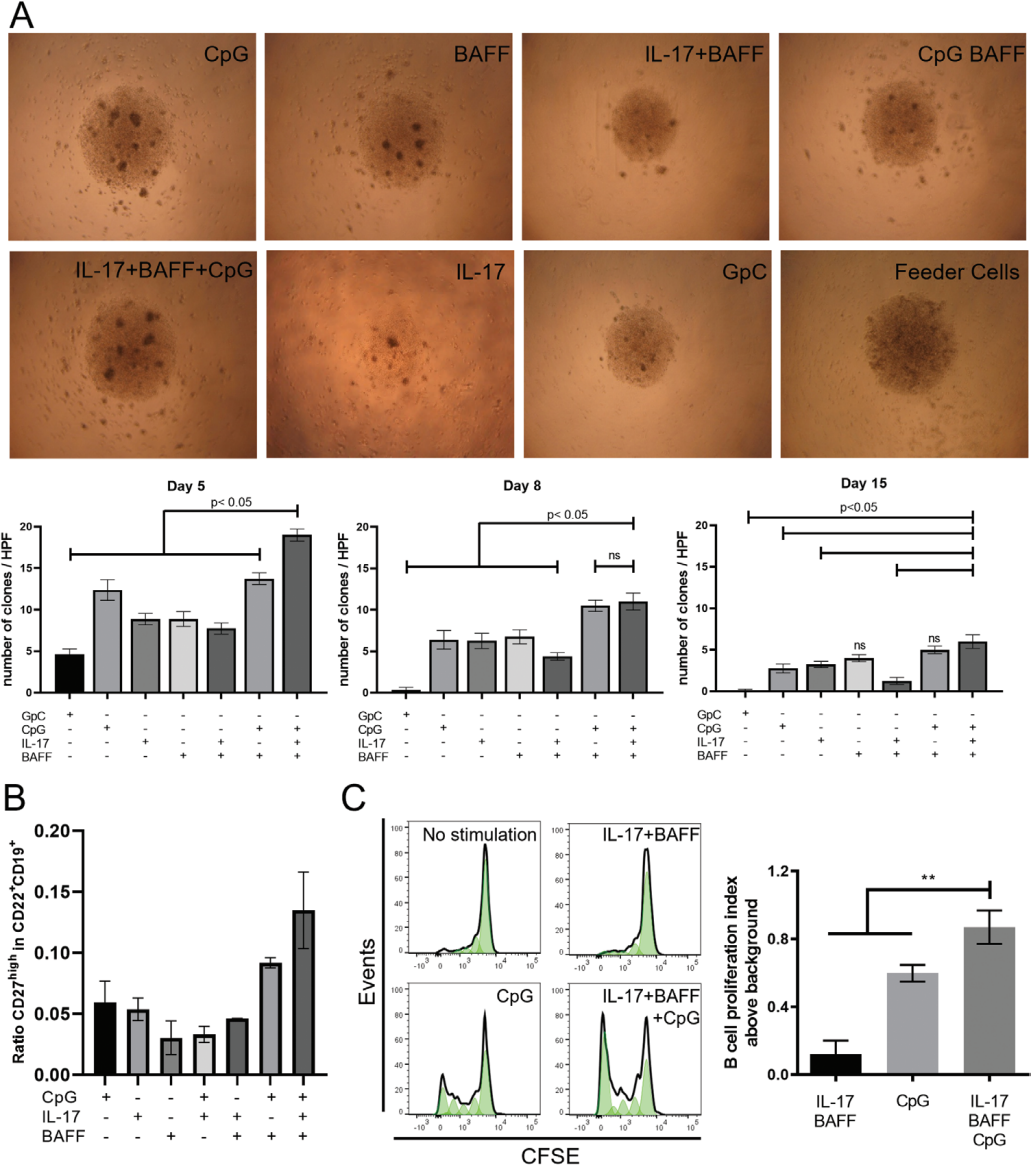
B cell activation with IL-17+BAFF+CpG led to colony formation, proliferation, and proportional increase in memory B cells. (**A**) Light microscopy images (top, representative images taken on day 5) and quantitative analysis per high power field (HPF) (bottom) of colony formation on days 5, 8, and 15 in *ex vivo* B cell cultures using different stimuli. Stimulation with IL-17+BAFF+CpG led to a larger number of colonies compared against all other conditions on day 5 (*n* = 2 independent experiments analysing 10 wells/condition) and compared to conditions lacking BAFF on day 8 and day 15. (**B**) IL-17+BAFF+CpG after 10 days led to a higher proportion of CD27^high^ in CD22^+^CD19^+^ B-cell cultures compared to other combination stimuli. (**C**) CFSE staining of live B cells (CD19^+^DAPI^-^) show a higher fold increase in the proliferation index of IL-17+BAFF+CpG stimulated B cells compared to CpG (*P* = 0.04) (or IL-17+BAFF [*P* = 0.02]) on day 5 (*n* = 5 performed in two independent experiments).

We evaluated whether stimulation of B cells with each IL-17, BAFF, or CpG, individually or combined, could support cell survival *ex vivo*. Co-culturing B cells in the presence of IL-17, BAFF, and CpG (IL-17+BAFF+CpG) yielded superior colony formation compared to other conditions on day 5 (compared with CpG: *P* = 0.0016; IL-17: *P* = 0.0002; BAFF: *P* = 0.0002; GpC ODN (GpC as control for CpG): *P* = 0.0002; IL-17+BAFF: *P* = 0.0002; CpG+BAFF: *P* = 0.0005; *n* = 2 independent experiments from two human healthy volunteers, 10 wells/volunteer) (Fig. 1A). On day 8, IL-17+BAFF+CpG continued to support a significantly higher number of colonies per well (compared with, CpG: *P* = 0.0064; IL-17: *P* = 0.005; BAFF: *P* = 0.0098; GpC: *P* = 0.0002; IL-17+BAFF: *P* = 0.0005). Significantly higher number of clones per well in IL-17+BAFF+CpG stimulated cultures were measured on day 15 compared to cells stimulated with: GpC: *P* = 0.0001; IL-17: *P* = 0.019; IL-17+BAFF: *P* = 0.0008. However, on day 15, IL-17+BAFF+CpG showed clonal formation similar to cells cultured with BAFF (*P* = 0.0645) or with CpG+BAFF (*P* = 0.3156).

**Fig. 2:**
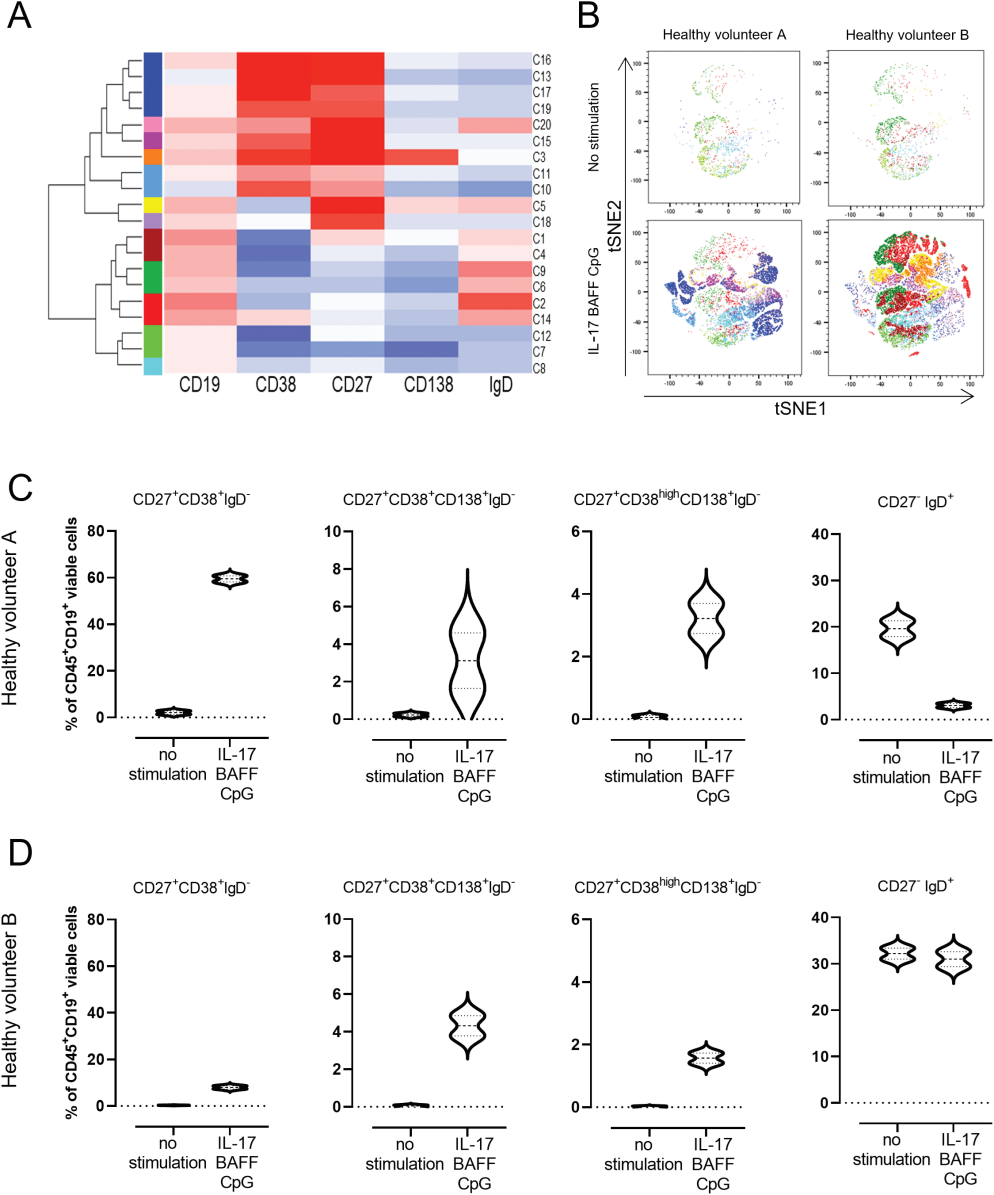
*Ex vivo* B-cell activation with IL-17+BAFF+CpG led to increased mature B-cell phenotypes. (**A**) Hierarchical clustering and heatmap of five B-cell markers (CD19, CD38, CD27, CD138, and IgD) show clustering of *ex vivo* cultured B-cell populations according to marker expression levels. Normalized Z-scores are displayed as colorimetric scale from blue to red. (**B**) tSNE maps of viable B (CD45^+^CD19^+^) cells from two healthy volunteers cultured *ex vivo*: unstimulated cells (top panel); IL-17+BAFF+CpG stimulated cells (bottom panel). (**C–D**) Violin plots showing B-cell populations after stimulation with IL-17+BAFF+CpG compared with no stimulation after 14 days in culture (data from two healthy volunteers, each experiment was run in duplicate). Circulating B cells were extracted from a healthy volunteer with detectable circulating autoantibodies against FOLR (Healthy volunteer A; C) and from a healthy volunteer with no detectable serum autoantibodies against FOLR (Healthy volunteer B; D).

To further understand these B-cell populations, we performed flow cytometric analysis of CD27^high^ mature memory cells within CD19^+^CD22^+^ B cells after 10 days in culture. Stimulating with IL-17+BAFF+CpG favoured expansion of the CD27^high^ within the CD19^+^CD22^+^ B-cell compartment (data from three healthy volunteers) (Fig. 1B). To independently confirm that this IL-17+BAFF+CpG stimulus yielded higher proliferation compared with the previously published stimulation with CpG [6], we performed a proliferation assay (CFSE). We found higher proliferation of IL-17+BAFF+CpG-stimulated live CD19^+^DAPI^-^ B cells compared with CpG (*P* = 0.0079) or IL-17+BAFF (*P* < 0.0079) (Fig. 1C). Unsupervised flow cytometric analysis (t-distributed stochastic neighbour embedding [tSNE] algorithm) of B-cell cultures from two healthy volunteers showed 12 distinct clusters of *ex vivo* cultured B cells and B-cell expansion with IL-17+BAFF+CpG stimulation (Fig. 2A and B). Consistent with IL-17+BAFF+CpG likely supporting B-cell differentiation (Fig. 1B), subset analyses of *ex vivo*-cultured B cells from the two healthy volunteers confirmed that IL-17+BAFF+CpG may favour class-switched, memory as well as CD19^+^CD138^+^CD38^+^CD27^+^IgD^-^ (and their subpopulation of CD38^high^ expressing CD19^+^CD138^+^CD38^high^CD27^+^IgD^-^) plasmablasts [17], rather than naive (CD19^+^CD38^-^CD27^-^IgD^+^) B cells (Fig. 2C and D, *n* = 2 healthy volunteers; see Supplementary Fig. 1for gating strategy).

**Fig. 3:**
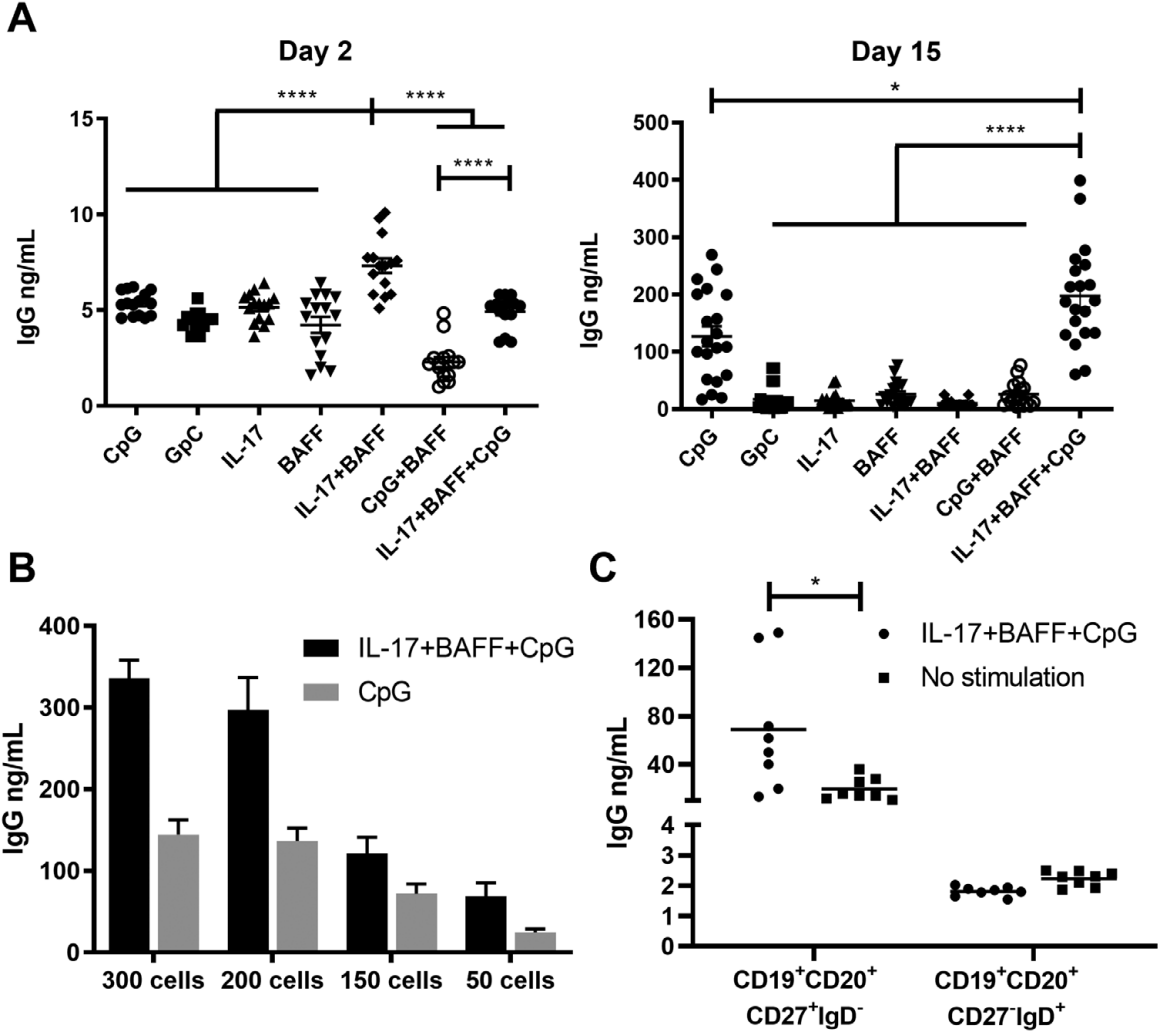
*Ex vivo* stimulation of human B cells increased IgG secretion. (**A**) IgG secretion in the supernatants of B cell cultures following activation with different stimuli showed IL-17+BAFF+CpG, and to a lesser extent CpG alone, yielded significantly higher IgG titres on day 15 (*n* = 2 independent experiments from two human healthy volunteers; 10 wells per experiment, of 200 B cells per well). (**B**) Increased IgG secretion in the presence of IL-17+BAFF+CpG compared to CpG at different plating densities of B cells (300, 200, 150 or 50 cells per well; representative experiment). (**C**) Stimulation with IL-17+BAFF+CpG (50 B cells per well) increased IgG secretion in CD19^+^CD22^+^CD27^+^IgD^-^ class-switched memory B cells but not in CD19^+^CD22^+^CD27^-^IgD^+^ mature naive B cells.

We then studied IgG secretion in B cell culture supernatants. Culture supernatants from B cells stimulated with IL-17+BAFF showed significantly higher IgG titres on day 2 compared to B cells cultured in other conditions (*n* = 9, *P* < 0.0001; Fig. 3A, left). On day 15, IL-17+BAFF+CpG stimulation, and CpG alone to a lesser extent, triggered the highest IgG titres compared with all other conditions (*P* < 0.05; *n* = 9) (Fig. 3A, right). When comparing the two most effective stimulation conditions for antibody production, namely IL-17+BAFF+CpG vs. CpG alone, at different B-cell plating densities (300, 200, 150, and 50 cells/well), IL-17+BAFF+CpG stimulation triggered superior IgG titres at all cell densities and levels of IgG were proportional to cell density (Fig. 3B, *n* = 1 representative of three independent experiments). Furthermore, consistent with preferential expansion of differentiated B cells (Figs 1B and 2C and D), IL-17+BAFF+CpG stimulation triggered significantly higher IgG secretion by mature CD19+CD20+CD27+IgD- memory B cells but not by CD19+CD20+ IgD-CD27- B cells. As expected, IgG levels were very low in IgD+ cell cultures (Fig. 3C).

**Fig. 4:**
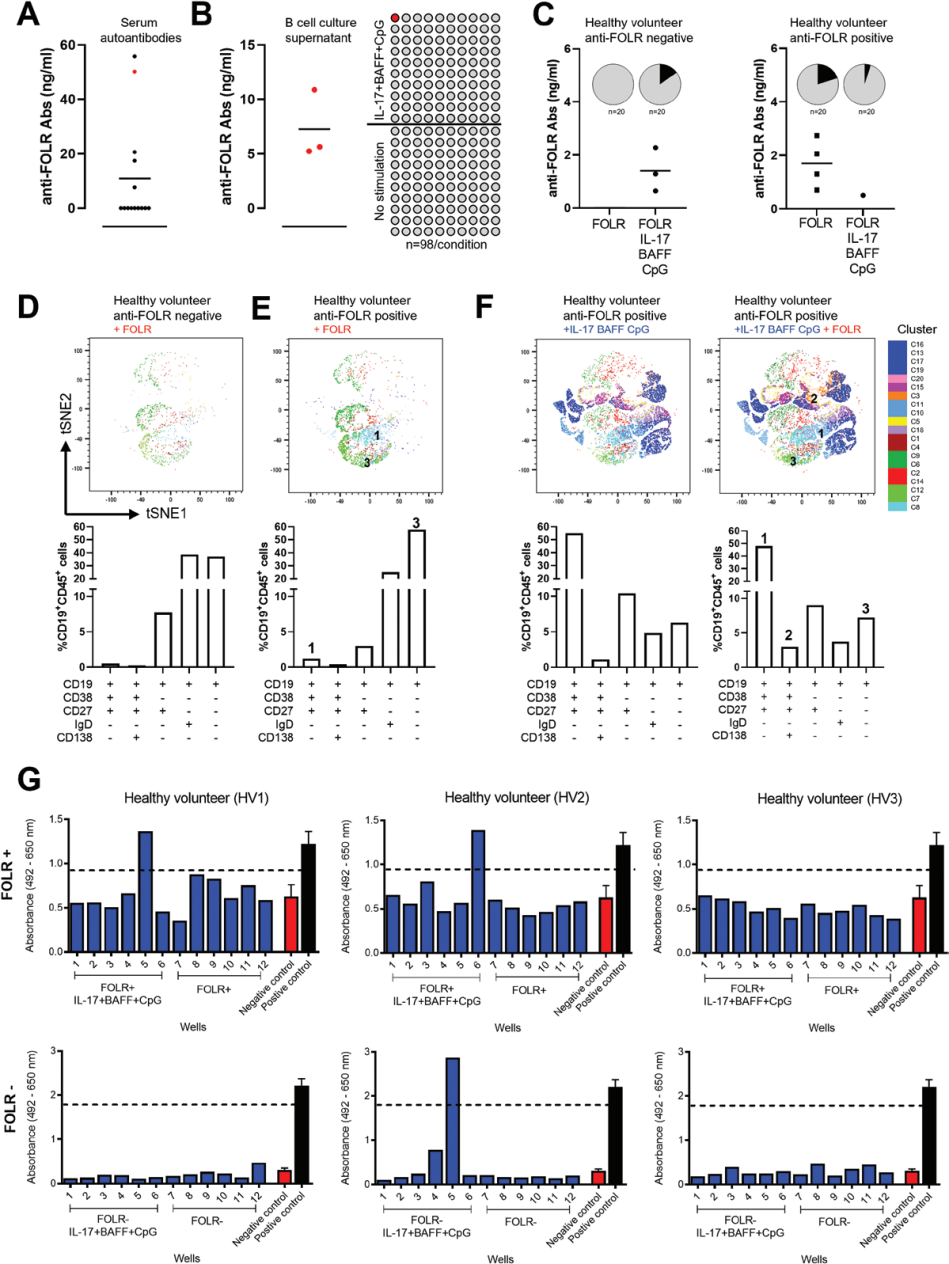
Tumour cell- and antigen-reactive antibodies can be detected in the supernatants of IL-17+BAFF+CpG-stimulated and antigen-stimulated B cells. (**A**) FOLR ELISA assays of serum samples from healthy volunteers (*n* = 15) show detectable autoantibodies against the tumour-associated antigen folate receptor alpha (FOLR) in five individuals. (**B**) FOLR ELISA assays of B-cell culture supernatants from a healthy volunteer with detectable serum anti-FOLR autoantibodies (red in A). In three independent experiments performed from the B-cell cultures of the same healthy volunteer, we tested *n* = 98 wells per independent experiment. This confirmed secretion of FOLR-specific IgG from ex vivo cultured B cells stimulated with IL-17+BAFF+CpG (*n* = 98 wells), but not from unstimulated B-cell cultures (*n* = 98 wells) (left: positive well confirmed in all three independent experiments performed from the same healthy volunteer; right: schematic of *n* = 98 wells per experiment). (**C**) B cells cultured with human recombinant antigen FOLR in the presence or absence of IL-17+BAFF+CpG led to anti-FOLR-specific antibodies in healthy volunteers with and without previously detectable serum autoantibodies (*n* = 2; 20 samples/condition, pie charts show proportion of positive cultures [black]). (**D–E**) Immunophenotyping (tSNE [top] and B-cell subset [bottom]) analysis of B cells from a healthy volunteer with no detectable serum anti-FOLR Abs (D), and from an individual with detectable serum anti-FOLR Abs (**E**), following *ex vivo* B-cell stimulation with recombinant FOLR. (**F**) Immunophenotyping (tSNE [top] and B-cell subset [bottom]) analysis of B-cell populations in a healthy volunteer following activation with IL-17+BAFF+CpG in the absence (left) or presence (right) of FOLR. (**G**) B cells cultured with IL-17+BAFF+CpG and the human recombinant antigen (FOLR) revealed secreted IgG antibodies binding to FOLR-expressing IGROV1 tumour cells. IGROV1 cell-based ELISA for the assessment of IGROV1-reactive IgG antibodies from culture supernatants of B cells from three healthy volunteers. B cells were stimulated with IL-17+BAFF+CpG and FOLR (top panel) or without FOLR (bottom panel). Assays were performed from culture supernatants collected on day 7. Each blue bar chart represents a supernatant sample from one B-cell culture well (blue). Controls (*n* = 3; mean ± SEM): Positive control anti-FOLR IgG, MOv18 clone, 400 ng/ml (black); Negative control, non-specific human IgG, 400 ng/ml (red). Threshold for IGROV1 reactivity was set at 75% the optical density (OD) value obtained for the positive control.

We next evaluated the potential antigenic reactivities of antibodies from *ex vivo*-cultured B cells. We detected circulating autoantibodies recognizing the tumour-associated antigen folate receptor alpha (anti-FOLR) in the sera from a cohort of healthy volunteers (*n* = 15, Fig. 4A). Using FOLR as a model antigen, we detected the presence of FOLR-specific antibodies in culture supernatants of B cells activated with IL-17+BAFF+CpG, and no detectable antibodies in supernatants of unstimulated B cells (*n* = 98 wells/condition, *n* = 3 independent experiments, Fig. 4B). Moreover, following stimulation with IL-17+BAFF+CpG in combination with recombinant FOLR, we detected anti-FOLR antibodies from cultured B cells of a healthy volunteer with no previously detected serum autoantibodies. In contrast, activation with FOLR in a healthy volunteer with previously detectable serum autoantibodies showed antibodies against FOLR following *ex vivo* stimulation of B cells with antigen alone (FOLR) and with FOLR in combination with IL-17+BAFF+CpG (*n* = 20 wells/condition, Fig. 4C). We then used hierarchical clustering to compare populations between FOLR-stimulated B cells in two individuals, one with and one without detectable serum FOLR autoantibodies. In the individual with previously detectable serum FOLR autoantibodies, we detected higher class-switched memory CD27^+^CD38^+^IgD^-^ B cells (Fig. 4E, population 1) and class-switched memory precursor CD27^-^CD38^-^IgD^-^ B-cell populations (Fig. 4E, population 3) (Fig. 4D and E). Furthermore, in B cells from an individual with previously detectable serum FOLR autoantibodies, we compared IL-17+BAFF+CpG-cultured cells with or without FOLR stimulation. These analyses showed that stimulation with IL-17+BAFF+CpG with FOLR favoured antibody-secreting plasma CD27^+^CD38^+^CD138^+^IgD^-^ cells (Fig. 4F, population 2) and, to a smaller extent, class-switched memory B-cell precursors (CD27^-^CD38^-^IgD^-^) (Fig. 4F, population 3 and tSNE maps). Simultaneous activation of B cells with the cancer-associated antigen Folate Receptor alpha (FOLR) together with IL-17+BAFF+CpG also allowed the detection of secreted antibodies recognizing FOLR-expressing cells (IGROV1) in individual B-cell cultures from two of the three healthy volunteers tested (IGROV1 cancer cell-based ELISA, Fig. 4G).

**Fig. 5:**
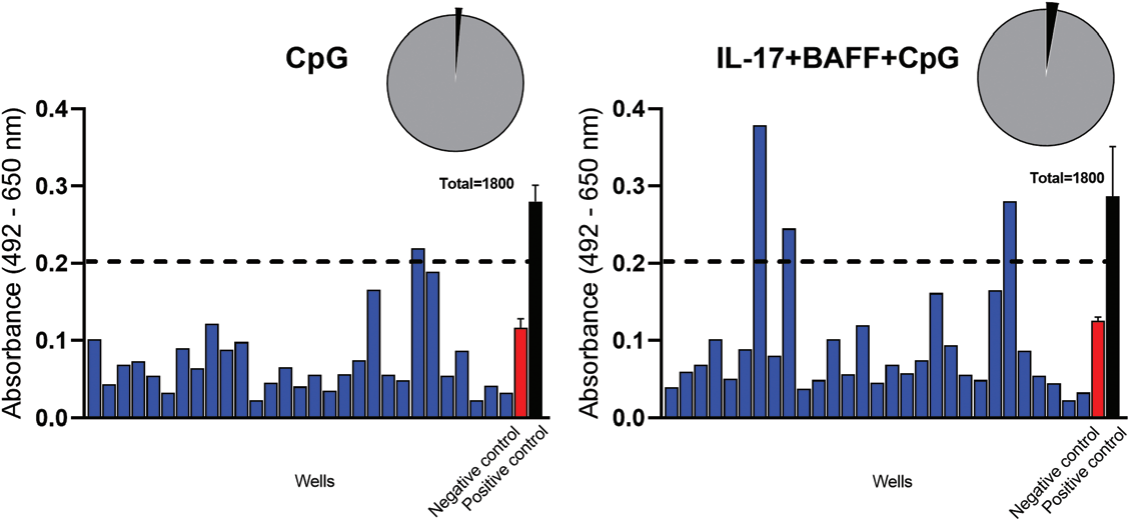
Tumour cell-reactive antibodies can be detected in the supernatants of IL-17+BAFF+CpG-stimulated B cells form melanoma patients. (**A**) A representative screen for tumour cell-reactive antibodies secreted after 10 days in supernatants of B-cell cultures from one of three patients with melanoma (Table 1 shows summary of data from three patients). Tumour cell-reactive antibodies were compared to a Negative control (non-specific human IgG, red bar) and a positive control (black bar, trastuzumab IgG, recognizing HER2/neu-expressing A375 cells) in the cell-based ELISA. Each blue bar represents a supernatant sample from one B-cell culture well. Samples showing specific binding to A375 melanoma cells (OD values > 75% of the positive IgG control) were considered reactive.

Next, using a cancer cell-based ELISA [18], we screened for tumour-reactive antibodies in B-cell cultures from three patients with malignant melanoma. This identified tumour cell-reactive antibodies from B cells stimulated with IL-17+BAFF+CpG in 51 of 1800 wells (2.8%) tested, and in 27 out of 1800 wells (1.5%) of B cells stimulated with CpG alone (Fig. 5, Table 1). No antibodies reacting with primary human melanocytes were detected from any of the B-cell cultures (Table 1). We then studied the reactivity of B cells from six patients with melanoma against tumour cell-associated antigens conjugated on fluorescent beads (Table 2). Individual B cells were likely to expand in response to IL-17+BAFF+CpG and the frequency of antigen-reactive B-cell selection increased following *ex vivo* culture compared to selection of freshly isolated B cells.

**Table 1.** Reactivity of melanoma patient-derived antibodies against a melanoma cell line and primary melanocytes

IL-17+BAFF+CpG B cell cultures							
Patient	Sex	Age	Stage	Cell type			
				Melanoma cells (A375)		Melanocytes	
				Mean fold increase above Negative control (95%CI)	Number of reactive cultures (total wells)	Mean fold increase above Negative control (95%CI)	Number of reactive cultures (total wells)
187	Male	64	IV	1.7 (1.5–2.2)	21 (600)	0.4 (0.3–0.5)	0 (600)
167	Male	80	IV	2.0 (1.9–2.1)	29 (600)	0.8 (0.8–0.9)	0 (600)
139	Female	55	IIIC	1.6 (1.3–1.9)	1 (600)	0.5 (0.4–0.6)	0 (600)
% Tumour-reactive cultures					2.8		0
CpG B cell-cultures							
Patient	Sex	Age	Stage	Cell type			
				Melanoma cells (A375)		Melanocytes	
				Mean fold increase above Negative control (95%CI)	Number of reactive cultures (total wells)	Mean fold increase above Negative control (95%CI)	Number of reactive cultures (total wells)
187	Male	64	IV	1.1 (0.7–1.5)	12 (600)	0.7 (0.6–1.3)	0 (600)
167	Male	80	IV	1.5 (1.4–1.6)	15 (600)	0.9 (0.8–1.1)	0 (600)
139	Female	55	IIIC	1.4 (1.2–1.9)	0 (600)	0.9 (0.9–1.0)	0 (600)
% Tumour-reactive cultures					1.5		0

Summary of B-cell culture supernatants from three melanoma patients screened for reactive antibodies against the human melanoma cell line A375 or against primary human melanocytes. Supernatants for screening of antibodies reactive to human cells in the cell-based ELISA were harvested after 10 days from B cells cultured with IL-17+BAFF+CpG or CpG alone. Mean fold increase above the Negative control wells (95%CI) were calculated by dividing the optical density of the sample by the optical density of the Negative control (non-specific human IgG). Culture supernatants were considered tumour cell-reactive if absorbance values were > 75% those measured with the Positive control (trastuzumab IgG, recognizing HER2/neu) against the same target cells.

**Table 2.** IL-17+BAFF+CpG stimulation of B cells from patients with melanoma increases the frequency of identification of antigen-reactive B-cell clones

Patient	Stage	Day 0, Frequency (Freshly isolated and sorted cells out of total B cells)	Number of sorted cells (Day 0)	Day 7, Frequency (Freshly isolated and sorted cells out of total B cells)	Number of sorted cells (Day 7)
1	III	<1/31,000	3	<1/237	7
2	II	<1/9000	5	1/250	40
3	Ib	1/20,000	6	1/5000	7
4	IIa	<1/26,000	8	1/4200	2
5	IIb	1/30,000	25	1/25,000	20
6	IIIA	1/33,000	2	1/3,000	2

B-cell clones from patients with melanoma recognizing cancer cell-associated antigens conjugated on fluorescent beads were studied: a) from freshly isolated circulating B cells, selected by recognition of antigens conjugated on beads and sorted immediately; or b) after *ex vivo* culture with IL-17+BAFF+CpG for 7 days, prior to selection by binding to antigen-conjugated beads and single cell sorting. These suggest that these data suggest that individual B cell clones are likely to expand in response to innate stimuli and that the frequency of antigen-reactive clone selection was increased following *ex vivo* culture with IL-17+BAFF+CpG.

These findings suggested that *ex vivo* stimulation of B cells from patients with cancer could reveal antibodies which may recognize cancer cells.

## Discussion

Innate stimulation can activate B cells in a T-cell-independent manner. Here, we report that a combination of innate signals prolonged B-cell survival, proliferation, differentiation, and significant IgG secretion in culture supernatants *ex vivo* and aided the identification of tumour-associated antigen and cancer cell-reactive antibodies secreted by peripheral blood B cells of healthy volunteers and of cancer patients.

CpG motifs are patterns of bacterial DNA (e.g. 5ʹ-TCGTCGTTTTGTCGTTTTGTCGTT-3ʹ) recognized by B-cell-associated TLR-9 as non-self. TLR-9 engagement induces activation of pathways such as the MAP and the STAT3 kinase pathway, leading to B-cell activation, proliferation, and enhancement of the humoral response, which should ultimately result in secretion of pathogen-neutralizing antibodies [19]. We and others have previously showed that CpG can induce B-cell proliferation and increase antibody secretion [6, 18, 20].

Another survival signal for mature B cells, may come from BAFF stimulation via the classical as well as the alternative NF-κB pathways. Engagement with BAFF-BAFF-R may, in the presence of B-cell receptor (BCR) signalling, mediate class switching of B cells in the absence of any other signals. BAFF receptor mutations can lead to low numbers of, or absent class-switched memory B cells [21]. Stimulation of memory B cells *ex vivo* with BAFF alone on in combination with IL-17 can increase not only cell survival but also IgG production [22, 23]. In support, IL-17 has been reported to prolong B cell survival through activation of the transcription factor Act1 and of the anti-apoptotic protein Twist-1 [6]. Moreover, IL-17 was shown to enhance class switching *in vitro* more prominently than IFNγ and to directly induce antigen-experienced memory B-cell differentiation without secondary T-cell signals [24].

Previous studies in rheumatoid arthritis demonstrated that IL-17 in synergy with BAFF can bypass the requirement of TLR-induced B-cell proliferation and survival. Therefore, in light of these findings and our previous data showing enhanced IgG production by CpG-activated human B cells, we asked if additional engagement of IL-17 and BAFF could offer further strong survival signals to B cells. Since the interaction between T cells and B cells via CD40L is known to lead to B-cell activation and proliferation, here we wished further to evaluate the impact of T-cell-independent soluble innate activating molecules on human B cells [25].

We demonstrate that stimulation with BAFF led to increased colony formation, likely a result of prolonged B-cell survival *ex vivo*. Moreover, combining BAFF with IL-17 and CpG resulted in higher B-cell proliferation and increased IgG production compared to individual stimuli. These effects were mostly driven by stimulation of and higher IgG secretion by mature, differentiated and class-switched CD27+IgD- memory, plasmablast and plasma cells. This suggests that IL-17+BAFF+CpG mostly drives activation and expansion of and antibody production by mature B-cell subsets. Because of their powerful effects in inducing or augmenting B-cell activation as well as antibody production, polyclonal activator signals to B cells have been considered as potential strategies for enhancing the humoral arm of the immune response in the context of cancer immunotherapy [26]. The potential efficacy of such therapies may come from boosting mature, including cancer-reactive clones. It is also possible that innate stimulating and danger signals can prompt a broader polyclonal, autoreactive B-cell response that can trigger proinflammatory signals to confer a level of protection from cancer growth in either or both health and malignancy [27].

As we detected autoantibodies against the tumour-associated antigen FOLR in the serum of healthy volunteers, we used FOLR as a model antigen to evaluate whether innate stimulation in the presence or absence of antigen could trigger B cells to produce FOLR-reactive antibodies. B cells from an individual with no anti-FOLR serum antibodies could be stimulated *ex vivo* with IL-17+BAFF+CpG combined with FOLR to produce antibodies, including those recognizing FOLR. Furthermore, stimulating B cells with IL-17+BAFF+CpG combined with FOLR induced antibodies in culture supernatants that bound to FOLR-expressing cancer cells. This is consistent with previous research suggesting that IL-17 may drive production of specific antibodies in the presence of BCR activation [24]. Together, these might point to the presence of tumour-reactive humoral immune surveillance in non-malignant states. The clinical significance of these serum antibodies and of circulating cancer antigen-reactive B cells in conferring protection from potential tumour growth require further investigation.

Immunophenotyping B-cell populations following stimulation using hierarchical clustering analyses revealed increased differentiated and class-switched B-cell subsets with IL-17+BAFF+CpG regardless of additional FOLR stimulation. While FOLR stimulation in an individual with detectable serum autoantibodies induced class-switched memory precursor and class-switched memory B cells, FOLR combined with IL-17+BAFF+CpG induced class-switched memory B-cell precursors and antibody-secreting plasma cells [28–30]. These suggest that IL-17+BAFF+CpG with and without additional antigen stimulation may favour different subsets of class-switched, memory and plasma-like rather than naive B cells.

Furthermore, we identified tumour cell-reactive IgGs from *ex vivo* IL-17+BAFF+CpG-stimulated and to a lesser extent from CpG-stimulated B cells from patients with melanoma. Simultaneously, we did not detect IgG bound to primary melanocytes. This may suggest that boosted B-cell-derived antibodies were more likely to recognize malignant cells. These findings are consistent with our previously reported identification and *ex vivo* generation of tumour-reactive B-cell-derived antibodies from cancer patients, by employing limiting dilution, flow cytometric sorting and matched heavy and light chain variable region sequencing and antibody cloning [3, 18, 31]. While we and others have reported the presence of circulating tumour-reactive B cells and serum antibodies, humoral responses might not be sufficient to control tumour growth in patients with cancer [32, 33]. This may be due to several limitations, including cancer-associated immunosuppression, resulting in: (i) inadequate B cell activation signals to trigger mature antibody production, (ii) generation of antibodies with low affinity to antigens, and (iii) expression of immature and inactive antibody isotypes (e.g. IgM, IgG4, IgA1), influencing antibody engagement and activation of immune effector cells against cancer. Therefore, engineering of human B cell-derived antibodies may be necessary to improve their functionality, such as by conjugating with cytotoxic agents or by modifying Fc regions to enhance recruitment of immune effector cells [34].

In summary, our data indicate that IL-17+BAFF+CpG, alongside antigen stimulation, in individuals with and without detectable circulating antigen-specific antibodies, may support the survival of differentiated, memory and often antigen-educated B cell compartments and the production of antibodies by antigen-reactive B cells. *Ex vivo* stimulation may be applied for the study of B cells from cancer patients and their expressed antibodies, to address the challenge of dissecting low-frequency B-cell subsets. Since BAFF, IL-17, and TLR-9 agonistic stimuli act through several signalling cascades to induce B-cell growth, it is possible that their combinations may synergize to induce optimal activation of antigen-educated mature human B cells. Further studies can investigate the antigen specificity of these secreted antibodies and employ *ex vivo* stimulation to explore the contribution of humoral responses to anti-cancer immune surveillance.

## Supplementary Material

uxab005_suppl_Supplementary_Figure_S1

## Data Availability

Not applicable, all data are provided in the manuscript.

## Abbreviation

Ab: antibodies
BAFF: B cell activation factor
BCMA: B-cell maturation antigen
CFSE: Carboxyfluorescein Succinimidylester
CpG: DNA motif CpG ODN 2006
ELISA: Enzyme-linked immunosorbent assay
FOLR: folate receptor alpha
IL-17: interleukin-17
TACI: Transmembrane activator and CAML interactor
TLR-9: toll like receptor 9
