# Supplementary figures and images for: Innate stimulation of B cells ex vivo enhances antibody secretion and identifies tumour-reactive antibodies from cancer patients
Source: Clin Exp Immunol. 2021 Dec 5;207(1):84–94. doi: 10.1093/cei/uxab005 (PMC8802180; doi:10.1093/cei/uxab005)

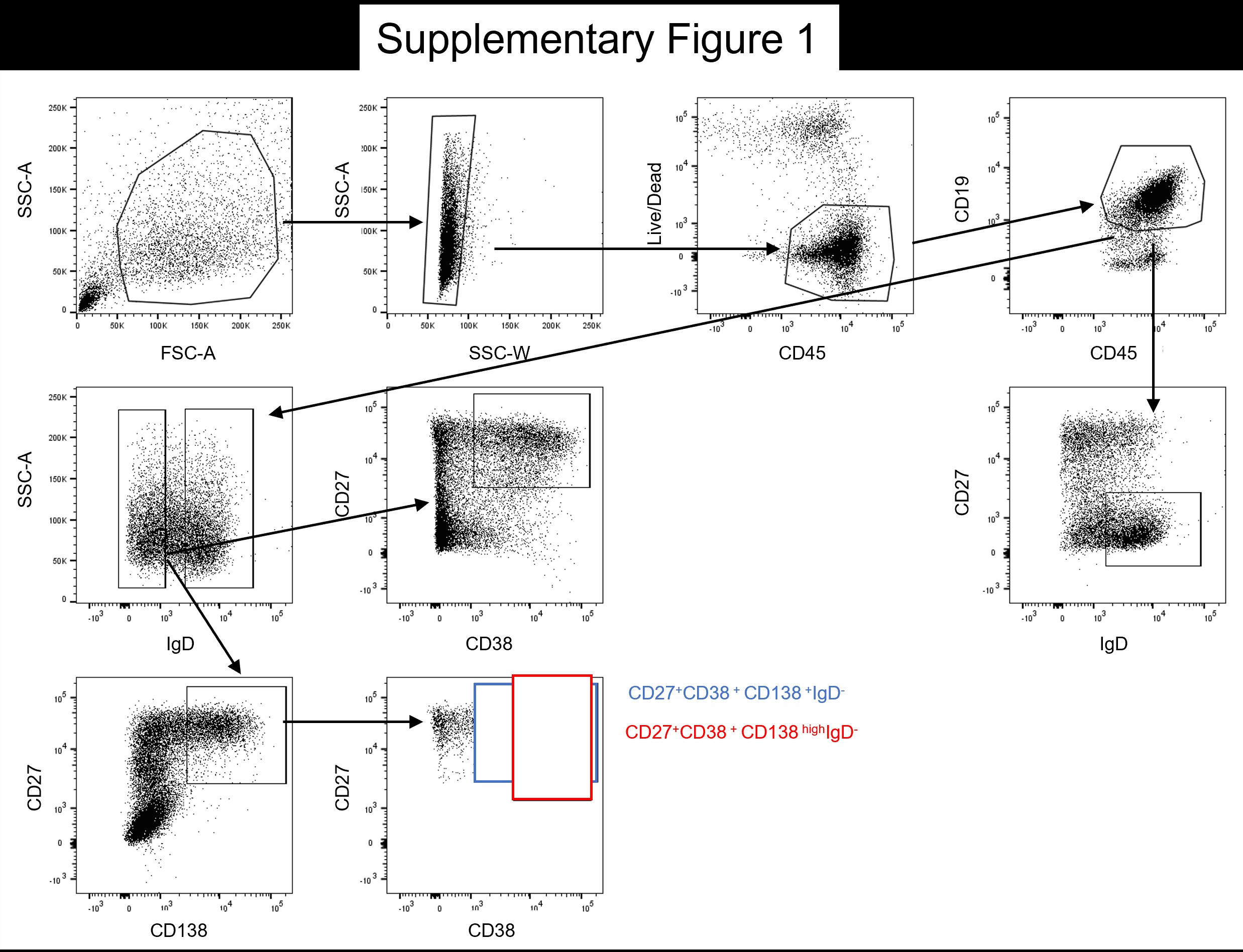

Supplement: uxab005_suppl_Supplementary_Figure_S1 [file uxab005_suppl_supplementary_figure_s1.jpeg]
